# Spatial Analysis of Changes in Cigarette Sales in Massachusetts and Bordering States Following the Massachusetts Menthol Flavor Ban

**DOI:** 10.1001/jamanetworkopen.2022.32103

**Published:** 2022-09-15

**Authors:** Samuel Asare, Anuja Majmundar, J. Lee Westmaas, Priti Bandi, Zheng Xue, Ahmedin Jemal, Nigar Nargis

**Affiliations:** 1 Tobacco Control Research, Surveillance & Health Equity Science, American Cancer Society, Atlanta, Georgia; 2 Population Science, American Cancer Society, Atlanta, Georgia; 3 Risk Factors & Screening Research, Surveillance & Health Equity Science, American Cancer Society, Atlanta, Georgia; 4 Surveillance & Health Equity Science, American Cancer Society, Atlanta, Georgia

## Abstract

This cohort study examines changes in cigarette sales in Massachusetts and its bordering states following a comprehensive ban on menthol flavor in Massachusetts in 2020.

## Introduction

In April 2022, the US Food and Drug Administration proposed a tobacco product standard that would prohibit menthol as a characterizing flavor in cigarettes nationwide.^1^ We previously reported that the comprehensive menthol flavor ban in Massachusetts, implemented in June 2020, was associated with reduced cigarette sales.^2^ One major limitation of the previous study was that it did not account for possible spillover in the 5 states bordering Massachusetts because of data unavailability for 3 of those states. The present study estimated changes in cigarette sales in Massachusetts and its bordering states associated with Massachusetts’ comprehensive menthol flavor ban to determine whether spillover in the bordering states offset the decreases in Massachusetts.

## Methods

This cohort study used state-level data on monthly aggregate cigarette sales volumes released for consumption from January 2017 to June 2021 based on required monthly filings by tobacco manufacturers and importers to the US Department of the Treasury. The monthly aggregate volumes of cigarette sales were converted into packs of cigarette (20 sticks) sales per capita based on state-level annual population data obtained from the US Census Bureau.

We used a spatial lag regression model specification (eMethods in the Supplement) to compare temporal changes in cigarette sales in Massachusetts and its bordering states before (January 1, 2017, to May 31, 2020) and after (June 1, 2020, to June 30, 2021) the comprehensive flavor ban with changes in 40 states and the District of Columbia (DC) that did not implement local or statewide menthol flavor bans. The model allowed for spatial dependence in cigarette sales between Massachusetts and its bordering states to obtain unbiased and consistent estimates.^3^ The study followed the Strengthening the Reporting of Observational Studies in Epidemiology (STROBE) reporting guideline. Because the study did not directly involve human participants, it did not require institutional review board approval or informed consent in accordance with the Common Rule.

All statistical tests were 2-sided, and *P* < .05, as calculated by *t* test, was considered to be statistically significant. Analyses were conducted using Stata, version 15.1 (StataCorp LLC).

## Results

Differences in cigarette prices and sociodemographic characteristics between Massachusetts and the comparison states were similar to those reported in our previous study.^2^ The spatial correlation estimate (ρ = −0.29; 95% CI, −0.54 to −0.04) indicates that changes in cigarette sales in Massachusetts were inversely associated with changes in cigarette sales in its bordering states (Table 1). Compared with the comparison states, monthly cigarette sales per 1000 persons decreased in Massachusetts by 350.02 packs (95% CI, −462.35 to −237.69 packs; *P* < .001) and increased in Massachusetts bordering states by 9.51 packs (95% CI, 2.72 to 16.30 packs; *P* = .007), for a net decrease of 340.51 packs (95% CI, −453.97 to −227.04 packs, *P* < .001) following the implementation of the ban (Table 1). Based on state-level annual population, total monthly cigarette sales declined in Massachusetts by approximately 2.45 million packs and increased in Massachusetts bordering states by approximately 0.13 million packs, for a net decrease of 2.32 million packs (Table 1). The estimates were robust after including New York (a bordering state with a local-level menthol flavor ban) and all states and the District of Columbia in the analysis (Table 1). The estimates from the difference-in-differences specification that did not account for spatial correlation in cigarette sales were consistently higher for Massachusetts and statistically insignificant for the bordering states (Table 2).

**Table 1.** Spatial Lag Model Estimates of Changes in Monthly Packs of Cigarette Sales Associated With Massachusetts’ Menthol Flavor Ban

	Comparison states are all states and the District of Columbia without local menthol favor ban; bordering states exclude New York	Comparison states are all states and DC without local menthol flavor ban; bordering states include New York	Comparison states are all other states; bordering states include New York
**Cigarette sales per 1000 persons** ^a^			
Changes in Massachusetts, No. of packs (95% CI)	–350.02 (–462.35 to –237.69)	–416.89 (–574.13 to –259.65)	–436.94 (–577.92 to –295.96)
Changes in the bordering states, No. of packs (95% CI)^b^	9.51 (2.72 to 16.30)	10.52 (0.53 to 20.50)	11.34 (2.99 to 19.69)
Net change, No. of packs (95% CI)	–340.51 (–453.97 to –227.04)	–406.38 (–567.77 to –244.98)	–425.60 (–568.82 to –282.38)
Spatial correlation coefficient (95% CI)	–0.29 (–0.54 to –0.04)	–0.23 (–0.49 to 0.02)	–0.27 (–0.53 to –0.02)
No. of observations	2484	2538	2754
**Total change in cigarette sales, in million packs^c^**			
Total changes in Massachusetts	–2.45	–2.92	–3.06
Total changes in the bordering states	0.13	0.36	0.38
Net change	–2.32	–2.57	–2.68

^a^ The estimates were obtained from a spatial lag model specification, described in the eMethods in the Supplement. The estimates in each column come from a separate regression. Models included mean cigarette price, state fixed effects controlling for time-invariant smoking characteristics, state-level time-varying factors (unemployment rate, age, sex, marital status, household income, education, race and ethnicity, and COVID-19 infection cases), and year-by-month fixed effects to account for time-invariant characteristics that are common in the fiscal year and seasonality in smoking. Models also included a spatial lagged dependent variable to allow cigarette sales in Massachusetts to be explained by the average cigarette sales in the bordering states and cigarette sales in Massachusetts bordering states to be explained by cigarette sales in Massachusetts. Standard errors were clustered within states.

^b^ The bordering states are Connecticut, New Hampshire, New York, Rhode Island, and Vermont.

^c^ Total changes in cigarette sales were obtained by multiplying the estimates for cigarette sales per 1000 persons by their respective average population data from 2020 to 2021.

**Table 2.** Difference-in-Differences Estimates of Changes in Monthly Sales of Cigarettes per 1000 Persons Associated With the MA Menthol Flavor Ban^a^

	No. of observations	Difference in packs per 1000 persons (95% CI)
**Change in MA vs comparison states**		
Comparison states are all other states and DC without local menthol favor ban and MA bordering states	2268	–553.96 (–653.37 to –454.54)
Comparison states are all other states and DC without local menthol favor ban	2538	–572.20 (–664.80 to –479.61)
Comparison states are all other states and DC	2754	–592.33 (–689.99 to –494.67)
**Change in bordering states** ^b^ ** vs comparison states**		
Comparison states are all other states and DC without local menthol favor ban and MA; bordering states excluded New York	2430	537.59 (–123.12 to 1198.30)
Comparison states are all other states and DC without local menthol favor ban and MA; bordering states included New York	2484	425.89 (–131.54 to 983.32)
Comparison states are all other states and DC without MA; bordering states included New York	2700	439.14 (–140.18 to 1018.46)

Abbreviations: DC, District of Columbia; MA, Massachusetts.

^a^ The estimates were obtained from a difference-in-differences model specification described in the eMethods in the Supplement. Each estimate comes from a separate regression. Models included mean cigarette price, state fixed effects controlling for time-invariant smoking characteristics, state-level time-varying factors (unemployment rate, age, sex, marital status, household income, education, race and ethnicity, and COVID-19 infection cases), and year-by-month fixed effects to account for time-invariant characteristics that are common in the fiscal year and seasonality in smoking. Standard errors were clustered within states.

^b^ The bordering states are Connecticut, New Hampshire, New York, Rhode Island, and Vermont.

## Discussion

The net decline in total cigarette sales after the Massachusetts comprehensive menthol flavor ban was consistent with a previously published study’s findings^2^ and demonstrates that initial increases in cigarette sales in the bordering states were not sustained.^4^ Limitations of this study are that online cigarette sales were not accounted for and that Massachusetts implemented other tobacco control policies that could affect the results. The findings demonstrate that the US Food and Drug Administration’s ban on menthol cigarettes may reduce overall cigarette consumption nationwide.

## Notes

Supplement.
eMethods.

eReferences
