# Supplementary material for: Spatial Analysis of Changes in Cigarette Sales in Massachusetts and Bordering States Following the Massachusetts Menthol Flavor Ban
Source: JAMA Netw Open. 2022 Sep 15;5(9):e2232103. doi: 10.1001/jamanetworkopen.2022.32103 (PMC9478773; doi:10.1001/jamanetworkopen.2022.32103)
Supplement: Supplement. — eMethods. eReferences [file jamanetwopen-e2232103-s001.pdf]

Supplemental Online Content

Asare S, Majmundar A, Westmaas JL, et al. Spatial analysis of changes in cigarette sales in Massachusetts and bordering states following the Massachusetts menthol flavor ban. *JAMA Netw Open*. 2022;5(9):e2232103. doi:10.1001/jamanetworkopen.2022.32103

eMethods.

eReferences

This supplemental material has been provided by the authors to give readers additional information about their work.

© 2022 Asare S et al. *JAMA Network Open*.

eMethods

Consider a standard difference-in-differences specification below:

$$Y_{st} = \tilde{\alpha}_0 + \tilde{\alpha}_1 MABAN_{st} + \tilde{\alpha}_2 APrice_{st} + \tilde{\boldsymbol{\beta}}\boldsymbol{X_{st}} + \boldsymbol{\gamma_s} + \boldsymbol{\gamma_t} + \mu_{st}, \qquad (1)$$

where Y_{st} = monthly sales of packs of cigarettes per 1000 persons in state s at time t. The monthly sales of packs of cigarettes per 1000 persons were measured using sales volumes obtained from the Alcohol and Tobacco Tax and Trade Bureau of the U.S. Department of the Treasury[1] divided by state-level annual population from the U.S. Census Bureau.[2,3] The variable $APrice_{st}$ represents the corresponding average price of cigarette packs (20-stick), $MABAN_{st}$ = indicator of the Massachusetts' menthol flavor ban at time t that equals 1 after May 2020 and 0 otherwise, $\boldsymbol{X_{st}} =$ vector of state-level time-varying factors sourced from the Bureau of Labor Statistics (unemployment rate),[4] Current Population Survey (population composition by age, sex, marital status, household income, education, and race/ethnicity),[5] and New York Times (COVID-19 infection cases that accounted for the confounding effects of the pandemic).[6] The vector of state fixed effects ($\boldsymbol{\gamma_s}$) accounts for the associations of state-specific time-invariant characteristics with cigarette sales. Similarly, the vector of year-by-month fixed effects ($\boldsymbol{\gamma_t}$) accounts for time-invariant characteristics that are common in the fiscal year and monthly seasonality in cigarette sales. The component ξ_{st} in equation (1) represents all unobserved state by time-varying factors that affect cigarettes sales. Standard errors were clustered within states.

The treatment state was Massachusetts. Also, the five bordering states of Massachusetts (Connecticut, New Hampshire, New York, Rhode Island, and Vermont) were

© 2022 Asare S et al. *JAMA Network Open*.

considered to be affected by the Massachusetts menthol flavor ban through cross-border purchases. The comparison states were all 40 states and the District of Columbia (DC) that did not implement local or state-wide menthol flavor bans. The comparison states excluded California, Colorado, Illinois, and Minnesota since some localities in these states implemented a local-level menthol flavor ban. We provided results for the case with and without New York in the bordering states. New York shares a border with Massachusetts but has a jurisdiction that implemented a local-level menthol flavor ban (i.e., in Manheim, NY, with a population size of approximately 3000). Finally, we provided results for a case where all non-bordering states of Massachusetts and the District of Columbia were included in the comparison states while accounting for the local-level menthol flavor ban. There were nondivergent trends in state-level sales of cigarette packs per 1000 persons in Massachusetts and comparison states during the period before Massachusetts' comprehensive menthol flavor ban.

One assumption of the difference-in-differences model specification in equation (1) is that cigarette sales in any two states (i.e., states i and j, for $i \neq j$) are independent (μ_{it} and μ_{jt} are uncorrelated, for $i \neq j$), suggesting that changes in cigarette sales in Massachusetts do not influence cigarette sales in its bordering states (Connecticut, New Hampshire, New York, Rhode Island, and Vermont). However, anecdotal evidence suggests increases in tobacco sales and tax revenues in bordering states of Massachusetts due to cross-border purchases of menthol cigarettes by Massachusetts state residents following its implementation of the comprehensive menthol flavor ban.[7,8] Additionally, one study has shown a temporary increase in cigarette sales in New Hampshire following the comprehensive menthol flavor ban in Massachusetts.[9] Therefore, our parameter of interest

in equation (1), $\tilde{\alpha}_1$, will be biased and inconsistent if the independence assumption is violated because of spillover effects, including cross-border purchases, in the bordering states of Massachusetts following its implementation of the comprehensive menthol flavor ban.

The parameter of interest in equation (1) may be biased and inconsistent even if two separate models were estimated, one for Massachusetts vs. the comparison states and the other for the bordering states vs. the comparison states, where the comparison states include all non-bordering states of Massachusetts and DC and without local or state-wide menthol flavor ban. The approach may lead to biased and inconsistent estimates due to an omitted variable bias (explained later) from a possible spatial dependence ("comovement") in cigarette sales between Massachusetts and the bordering states after the comprehensive menthol flavor ban.[10]

We followed the spatial econometrics literature to address this limitation by estimating a single equation that identified both changes in cigarette sales in Massachusetts and changes in cigarette sales in the bordering states of Massachusetts (i.e., spillover effect) of the Massachusetts comprehensive menthol flavor ban after accounting for the spatial dependence in cigarette sales between Massachusetts and the bordering states. Based on the literature, a spatial lagged dependent variable ($\sum_{s \neq j} W_{sj} Y_{jt}$), with the spatial correlation coefficient ($\boldsymbol{\rho}$),[10] was introduced into equation (1) as below:

$$Y_{st} = \rho \sum_{s \neq j} W_{sj} Y_{jt} + \alpha_0 + \alpha_1 MABAN_{st} + \alpha_2 APrice_{st} + \boldsymbol{\beta X_{st}} + \boldsymbol{\gamma_s} + \boldsymbol{\gamma_t} + \xi_{st}, \qquad (2)$$

© 2022 Asare S et al. *JAMA Network Open*.

where W_{sj} represents an $n \times n$ weighting matrix that indicates neighbor relationships, with $\boldsymbol{\rho}$ capturing the endogenous spatial interaction effects. Because we are only interested in the interaction between Massachusetts and its bordering states, we assumed that sales in all the other states are independent regardless of whether they share border or not (i.e., cigarette sales in state s do not affect sales in state j, for all $s \neq j$, and s and j are not Massachusetts or its bordering states). This assumption plausibly allowed us to capture only the spatial correlation in cigarette sales between Massachusetts and the average cigarette sales in its bordering states. Consequently, the weighting matrix was defined as $w_{25j} = w_{j25} = 1$ between Massachusetts (i.e., $s = 25$) and its bordering states (i.e., Connecticut *[j=9]*, New Hampshire *[j=33]*, New York *[j=36]*, Rhode Island *[j=44]*, and Vermont *[j=50]*) and 0 otherwise. Introducing the variable $\sum_{s \neq j} W_{sj} Y_{jt}$ in equation (2) allowed cigarette sales of cigarettes in Massachusetts to be explained by the average cigarette sales in the bordering states. Similarly, the model allowed cigarette sales in Massachusetts bordering states to be explained by cigarette sales in Massachusetts.

We demonstrate the relevance of allowing spatial dependence in cigarette sales between Massachusetts and it bordering states as follows. Suppose the true model has spatial correlation (i.e., $\rho \neq 0$) in cigarette sales between Massachusetts and its bordering states (see equation (2)) but a restricted model that assumes no spatial correlation (i.e., $\rho = 0$) in cigarette sales between Massachusetts and its bordering states (equation (1)) is estimated. Then $\tilde{\alpha}_1$ is an unbiased estimator of α_1 if $MABAN_{st}$ and $\sum_{s \neq j} W_{sj} Y_{jt}$ are uncorrelated.[11] In that case, using model specification (1) will give an estimate of the true association of the menthol ban with cigarette sales in Massachusetts if the menthol flavor ban was not associated with changes in cigarette sales in Massachusetts bordering states.

On the other hand, if $MABAN_{st}$ and $\sum_{s \neq j} W_{sj} Y_{jt}$ are correlated, then $\tilde{\alpha}_1$ is a biased estimator of α_1.In other words, if changes in cigarette sales in Massachusetts and border states co-vary and the Massachusetts menthol flavor ban was associated with changes in cigarette sales in the bordering states, then $\tilde{\alpha}_1$ is a biased estimator of α_1 because of omitted variable bias (i.e., $\sum_{s \neq j} W_{sj} Y_{jt}$ is omitted from equation (1)).[11]

We estimated the model in equation (2) using "xsmle" Stata command (STATACorp LLC), which can provide both fixed effect and random effect estimates. We estimated both fixed effect and random effect estimates and performed the Hausman specification test to select the appropriate estimation strategy. The Hausman specification test was used to test the null hypothesis of equality between fixed effect and random effect model estimates against the alternative hypothesis of the random effect model estimates being inconsistent. The Hausman test results for all three cases rejected the null hypothesis of equality in the estimates between the fixed effect and random effect models. Therefore, we reported only the fixed effect estimates for interpretation.

eReferences

1. Statistical report - tobacco. United States Department of Treasury, Alcohol and Tobacco Tax and Trade Bureau. Accessed July 28, 2021. https://www.ttb.gov/tobacco/tobacco-statistics

2. U.S. Census Bureau. State Population Totals: 2010-2019. The United States Census Bureau. Accessed June 2, 2021. https://www.census.gov/data/tables/time-series/demo/popest/2010s-state-total.html

3. US Census Bureau. State Population Totals and Components of Change: 2020-2021. Census.gov. Accessed January 28, 2022. https://www.census.gov/data/tables/time-series/demo/popest/2020s-state-total.html

4. Bureau of Labor Statistics. Civilian unemployment rate. Accessed April 13, 2021. https://www.bls.gov/charts/employment-situation/civilian-unemployment-rate.htm

© 2022 Asare S et al. *JAMA Network Open*.

5. Census Bureau. Basic Monthly Current Population Survey. The United States Census Bureau. Accessed January 5, 2022. https://www.census.gov/data/datasets/time-series/demo/cps/cps-basic.html

6. nytimes/covid-19-data. Published online July 12, 2021. Accessed July 12, 2021. https://github.com/nytimes/covid-19-data

7. Boesen U. Massachusetts Flavored Tobacco Ban: No Impact on New England Sales. Tax Foundation. Published February 3, 2022. Accessed May 3, 2022. https://taxfoundation.org/massachusetts-flavored-tobacco-ban-sales-jama-study/

8. Menthol-tobacco prohibition: Ban or just Band-Aid? Boston Herald. Published January 28, 2021. Accessed May 13, 2022. https://www.bostonherald.com/2021/01/28/menthol-tobacco-prohibition-ban-or-just-band-aid/

9. Kingsley M, McGinnes H, Song G, Doane J, Henley P. Impact of Massachusetts' Statewide Sales Restriction on Flavored and Menthol Tobacco Products on Tobacco Sales in Massachusetts and Surrounding States, June 2020. *American Journal of Public Health*. 2022;112(8):1147-1150.

10. Elhorst JP. Specification and Estimation of Spatial Panel Data Models. *International Regional Science Review*. 2003;26(3):244-268. doi:10.1177/0160017603253791

11. Wooldridge JM. Introductory Econometrics: A Modern Approach. (Sixth Ed. Pp. 78-79). Cengage learning; 2015.

© 2022 Asare S et al. *JAMA Network Open*.
